# Editorial: Psychosocial work environment during the COVID-19 pandemic

**DOI:** 10.3389/fpubh.2023.1272290

**Published:** 2023-09-01

**Authors:** Maria Malliarou, Aristomenis Kotsakis

**Affiliations:** ^1^Department of Nursing, School of Health Science, University of Thessaly, Larissa, Greece; ^2^Department of Management Science and Technology, School of Economics and Business Administration, University of Patras, Patras, Greece

**Keywords:** psychosocial, COVID-19, work, environment, risk

In our present Research Topic, “*Psychosocial Work Environment During the COVID-19 Pandemic*”, the number of articles that were published was 16, with a total of 4,660 downloads to date. Our topic investigates the cross-sectoral and multidimensional impact of COVID-19-related work environment changes on employees' psychosocial wellbeing. Furthermore, it investigates the psychosocial mechanisms explaining these effects in several countries and across different service industries using a “voice of the employee” approach. It includes the investigation of both direct and indirect impacts of COVID-19 on employees' psychosocial wellbeing. All relevant research findings are documented by an in-depth analysis of all primary research data, aiming to investigate and isolate the main psychosocial cause-and-effect mechanisms explaining these effects.

We strongly believe that our current Research Topic will act as an added-value component in the international psychosocial risk management research platform for exchanging cross-sectoral and cross-country practical and scientific knowledge between scientists and practitioners. In addition to the above, both strategic HR managers and policymakers could take into consideration our Research Topic findings for the implementation of “evidence-based” and “employee-centric” management interventions and policymaking in the near future.

The effect of working environment conditions on the health and safety of workers has been the subject of several scientific studies (1–3). Psychosocial risks refer to factors that can potentially cause psychological or physical harm to workers. Such factors may concern aspects of the planning, organization, and management processes of work, a lack of supportive relationships, job insecurity, or even the culture of a company (4).

Psychosocial risks arise from the problematic planning, organization, and management of work, as well as from an unhealthy social context of work, and may lead to negative psychological, physical, and social outcomes such as work stress, burnout, or depression (5). In addition to mental health disorders, workers suffering from prolonged stress are at risk of experiencing serious physical health problems, such as cardiovascular diseases or musculoskeletal problems (6). At the organizational or business level, negative consequences can include poor overall work performance, increased absenteeism, and increased accident rates and injuries (7).

The outbreak of COVID-19 had a great impact on employees' daily work and psychology, and many frontline personnel sacrificed their own wellbeing (Jiang et al.). More specifically, the pandemic has placed an additional burden on already strained healthcare systems worldwide, intensifying the responsibility of healthcare workers. Before the COVID-19 pandemic, previous studies had shown that adverse workplace factors were associated with the likelihood of developing mental health disorders among healthcare workers.

A high prevalence of functional gastrointestinal disorder (FGID)-related symptoms was observed among healthcare workers without a history of FGID during the period when they were involved in the fight against COVID-19 (Zhang et al.). This study was one of the articles cited in the Research Topic “*Psychosocial Work Environment During the COVID-19 Pandemic*”.

According to Muller et al. (8), individual, interpersonal, and organizational factors are recognized as workplace issues that have a negative influence on the mental health of health professionals, while workplace conflicts also have a negative influence. Workplace factors such as support in the workplace and health/safety in the workplace instead of coronavirus-related risks seem to be able to predict not only the present stress level but also the stress level over the long term. It was a really important finding that, during the COVID-19 pandemic period, in order to relieve the high stress of healthcare workers, organizational-level approaches should have been implemented, especially measures designed to enhance support and health/safety in the workplace, according to the Xiong et al..

Gu et al. proposed that policymakers and nursing administrators should pay close attention to the work stress of frontline nursing professionals because taking active and effective interventions and offering psychological support will help them to have a positive mindset. At the governmental level, occupational psychosocial risks should be included in the scope of OSH, including regulations, policies, and standards. At the organizational level, administrators are encouraged to work on preventing and controlling psychosocial risks and promoting mental health in workplaces. At the individual level, healthcare workers might increase awareness through universal training in psychosocial risk coping strategies.

In the context of the COVID-19 pandemic, employees were facing both the stress of their work commitments and the stress caused by the virus. Leaders communicating with employees about their physical and mental health was found to be of great importance as it could make employees feel that the organization is not only concerned about their work performance but also attaches importance to their health and safety (Xiong et al.), which promotes organization-based self-esteem and work engagement (9). One of the most cited articles on our topic is the article written by Tang et al., which suggests that managers should formulate policies and strategies to ensure and improve the interests and wellbeing of nurses and improve the practice environment to protect the sense of security of nurses, which is helpful to increase work engagement and reduce turnover intention. The safe communication of leaders makes employees feel that they are valued and useful in the organization, and they then tend to display more beneficial behaviors at work (10). It is crucial for leaders to provide timely psychological support to employees through communication (11, 12).

## Author contributions

MM: Writing—review and editing. AK: Writing—original draft.
